# Optimal blood pressure for the minimum all-cause mortality in Chinese ESRD patients on maintenance hemodialysis

**DOI:** 10.1042/BSR20200858

**Published:** 2020-08-13

**Authors:** Tao Wang, Yang Li, HaiBo Wu, Hua Chen, Yan Zhang, HuiMin Zhou, Hang Li

**Affiliations:** 1 Department of Science and Education, HeBei General Hospital, ShiJiaZhuang 050051, P.R. China; 2 Department of Oncology, HeBei General Hospital, ShiJiaZhuang 050051, P.R. China; 3 Department of Cardiology, HeBei General Hospital, ShiJiaZhuang 050051, P.R. China; 4 Department of Dermatology, The Fourth Hospital of HeBei Medical University, ShiJiaZhuang 050011, P.R. China; 5 Department of Endocrinology, The First Hospital of HeBei Medical University, ShiJiaZhuang 050000, P.R. China; 6 Department of Nephrology, Peking Union Medical College Hospital, Beijing 100010, P.R. China

**Keywords:** blood pressure, cardiovascular death, end stage renal disease, maintenance hemodialysis, mortality

## Abstract

Blood pressure (BP) is a known prognostic marker for mortality in patients on maintenance hemodialysis (MHD). However, definition of the BP and its optimal values vary essentially among different MHD populations. Our purpose was to clarify these important clinical parameters in a Chinese MHD cohort. Accordingly, we reviewed the available records of patients on regular MHD during the past 10 years and made a comparison between the deceased (*n*=81) and survival ones (*n*=131). Multiple logistic regression and Kaplan–Meier survival analysis were used to examine the effect of BP on mortality and long-term survival, respectively. The all-cause mortality in our patients was 38.2%, in which 49.4% was from cardio-cerebrovascular deaths. Using the multiple logistic regression, we found that the sitting (the same definition hereafter) pre-dialysis systolic BP (SBP) was significantly associated with both the all-cause mortality and cardio-cerebrovascular deaths exclusively in patients of 60–80 years. Moreover, a pre-dialysis SBP of 140–160 mmHg in these patients had the minimum all-cause mortality (23.5%) against that conferred by either a lower (42.1%) or higher SBP value (61.5%). This observation was further confirmed by the Kaplan–Meier survival analysis. As fresh gain to the practice of hemodialysis, our report revealed that BP worked in a time-dependent way among a Chinese MHD cohort and highlighted a U-shaped association between the pre-dialysis SBP and all-cause mortality. These findings may hence help to obtain optimal BP control for better survival and lend some prognostic insight into mortality in these MHD patients.

## Introduction

The incidence of cardiovascular events has increased significantly with the deterioration of renal function in such a way that it surged from 2.1% annually to 36.6%, when estimated glomerular filtration rate decreased from more than 60 to less than 15 ml/min/1.73 m^2^ [1]. In a 2013 report [2], 80.0% of the 0.4 million maintenance hemodialysis (MHD) patients in the United States were found to have cardiac diseases. Reportedly, cardio-cerebrovascular diseases (CVDs) accounted for 52.8% of all-cause mortality in MHD patients, followed by infectious diseases (21.6%) and malignancies (16.0%) [3].

Hypertension has long been recognized as one of the traditional CVD risk factors in these patients [4] and blood pressure (BP) is believed to be a prognostic marker for mortality [5]. However, there was remarkable disparity regarding the way of BP measurement in and its effect on the said prognostic significance. It was known that category (ambulatory, pre- or post-dialysis), measuring methods (supine, sitting or standing) and optimal values of the BP varied essentially among different MHD populations [6]. In this regard, mortality predictor was intradialysis hypotension as orthostatic systolic BP (SBP) of 110–119 mmHg in Japanese MHD patients [7]. In white and black Americans, it was either seated pre-dialysis or post-dialysis SBP of less than 120 mmHg, or ambulatory BP of 134–146 mmHg [8–10]. By comparison, BP was found to have no association with the cardiovascular mortality in French and Italian [11,12].

It is clear that BP manifested ethnic heterogeneity in predicting the prognosis among the MHD patients, yet most pertinent knowledge was based on or extrapolated from studies that excluded these very patients [2]. Indeed, we found a decade ago that results of genetic study in the Japanese could not be readily extended to other populations [13]. We therefore examined the effect of BP on all-cause mortality and CVD deaths in a Chinese MHD cohort. As such, we in this investigation found that a pre-dialysis SBP of 140–160 mmHg had the minimum all-cause mortality in patients of 60–80 years and these findings could be easily applied to those ethnically and geographically alike.

## Materials and methods

### Study population

In this retrospective cohort study, we reviewed the available records of the MHD patients deceased between 2009 and 2018 (*n*=81), and matched them against those of the survival ones (*n*=131), during the same period with comparable MHD vintage. These patients received three weekly sessions of hemodialysis for >6 months under the then KDIGO guidelines [14], using bicarbonate-based dialysate and polysulfone membrane dialyzers. They were at least 18 years old when initiated the MHD, without history of prior renal transplantation, absent from significant blood loss and free of surgery within 1 year. α-recombinant human erythropoietin and iron sucrose were given according to the same guidelines. Lifestyle factors, medical history and dialysis prescription were dated back by our team members. The study was approved by our institutional review board and all patients or the immediate kin gave written informed consent.

### BP measurement

We empirically selected the pre-dialysis BP which had been the prime focus in our long-term practice [15]. Further, the values were those taken before the midweek dialysis session, where volume withdrawal was set according to clinical standard on the basis of the personal dry-weight [16]. More precisely, seated pre-dialysis BP were measured prior to the start of the session by means of automatically inflated cuffs on the non-access arm using a digital monitor attached to each hemodialysis machine [10]. All the available BP values of each individual were eventually averaged. In particular, the last BP value was the one taken at the next-to-last hemodialysis session before the event and admission in case of sudden death or hospital demise, respectively.

### Laboratory tests

Other pertinent variables were also averaged based on the results of periodic dialysis evaluations. In this regard, venous blood was usually collected before the second hemodialysis session of the week after overnight fasting. Hemoglobin (Hb) concentration was acquired using Sysmex KX-21N hematology analyzer (Sysmex Corporation, Kobe, Japan). Serum biomarkers were measured by using Beckman Coulter AU5800 automatic biochemical analyzer (CA, U.S.A.). Ferritin was obtained by the use of enzyme-linked immunoassays (Monobind Inc, Lake Forest, CA). Transferrin saturation (TSAT, %) was calculated by dividing the serum iron concentration by the total iron binding capacity and multiplying the result by 100, to obtain a percentage. Intact parathyroid hormone (iPTH) was determined by commercially available kit (Elecsys, Roche Diagnostics GmbH, Germany). Kt/V of the hemodialysis was derived from the well-established KDOQI equation. Finally, the all-cause mortality and CVD deaths were defined as previously deliberated [8].

### Statistical analysis

Statistical analyses were performed using SPSS version 17.0 (SPSS, Chicago, IL, U.S.A.). All data used in the analysis were normally distributed as the significantly skewed ones were square root-transformed. Student’s *t* test and the chi-square test were used for comparing continuous and categorical variables between groups, respectively. Multiple logistic regression and Kaplan–Meier survival analysis were then respectively used to examine the effect of BP on mortality and survival. Two-sided *P*<0.05 was considered statistically significant.

## Results

### Risk factors for the studied subjects

The all-cause mortality in our patients was 38.2%, in which 49.4% was due to CVD deaths. Between the deceased and surviving patients, there were no differences with regard to the gender composition, age, time of dialysis, body mass index, Hb concentration, lipid profiles, albumin, electrolytes and hemodialysis-associated factors including the Kt/V, iPTH and TSAT (Table 1). Notably, the deceased patients were on the MHD for 40.3 ± 13.5 months, whereas the survival ones were for 46.4 ± 21.0 months. In comparison with the survival ones, however, the deceased patients had significantly higher pre-dialysis SBP but not the diastolic BP (DBP), high sensitivity C-reaction protein (hs-CRP), ferritin content and more diabetic, whereas the pre-dialysis serum creatinine (Scr) was lower. Also with a detectable tendency, fasting blood sugar (FBS) and total cholesterol appeared to be higher and lower, respectively. Rather unexpected, the ensuing multiple logistic regression did not support the pre-dialysis SBP as a significant determinant, prompting us to consider the existence of interfering factor.

**Table 1:** Demographic and clinical profile of the study cohorts with all-cause mortality

	Deceased	Surviving
*n*	81	131
Gender (male/female)	46/35	69/62
Age (y)	65.9 ± 15.9	61.0 ± 15.0
Time on dialysis (m)^‡^	40.3 ± 13.5	46.4 ± 21.0
Body mass index (kg/m^2^)	22.9 ± 2.2	23.1 ± 3.4
Pre-dialysis SBP (mmHg)	143.2 ± 32.7	137.4 ± 20.8*
Pre-dialysis DBP (mmHg)	79.0 ± 15.9	76.3 ± 18.0
Hb concentration (g/l)	87.2 ± 22.3	97.2 ± 9.7
Pre-dialysis Scr (μmol/l)‡	595.3 ± 222.3	808.2 ± 325.9*
Fasting plasma sugar (mmol/l)^‡^	6.4 ± 4.1	5.5 ± 3.6†
hs-CRP (mg/l)^‡^	18.8 ± 13.9	6.0 ± 3.8*
Total cholesterol (mmol/l)	3.79 ± 1.06	4.16 ± 1.22†
High density lipoprotein (mmol/l)	1.00 ± 0.37	1.05 ± 0.40
Low density lipoprotein (mmol/l)	2.44 ± 0.89	2.82 ± 0.94
Triglycerides (mmol/l)^‡^	1.31 ± 0.74	1.50 ± 1.07
Albumin (g/l)	31.7 ± 6.0	36.4 ± 5.1
Kt/V	1.30 ± 0.21	1.27 ± 0.28
Potassium (mmol/l)	4.6 ± 0.9	4.8 ± 0.7
Calcium (mmol/l)	2.1 ± 0.3	2.1 ± 0.2
Phosphate (mmol/l)	1.7 ± 0.6	1.8 ± 0.7
Parathyroid hormone (pg/ml)^‡^	40.4 ± 20.8	45.8 ± 22.0
Ferritin (ng/ml)^‡^	354.1 ± 95.0	316.7 ± 231.7*
TSAT (%)	27.8 ± 12.2	27.5 ± 10.6
Diabetes mellitus (%)	46.9	32.1*

Results are given as mean ± SD. Differences among the cohorts were examined by the *t* test or χ^2^ test when deemed appropriate.

* *P*<0.05.

† 0.05<*P*<0.10.

‡ Log-transformed values used in the analysis.

### Risk factors for patients of 60–80 years

**Figure 1:**
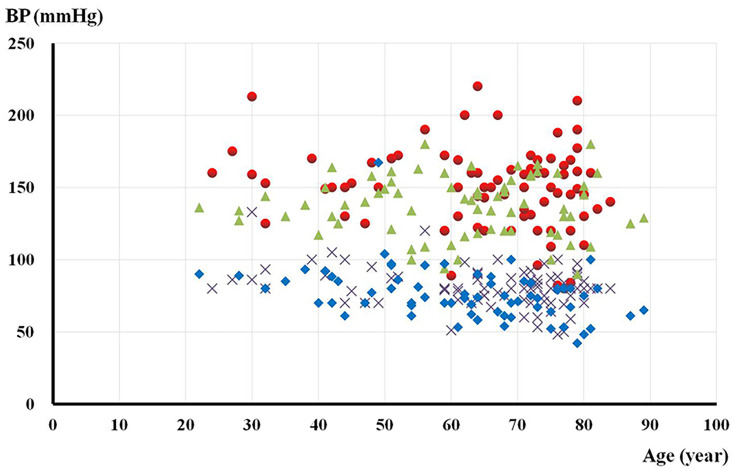
Systolic and diastolic pre-dialysis BP of the individual MHD patients across age Triangle and diamond symbols: systolic and diastolic pre-dialysis BP of the surviving patients, respectively. Round and cross symbols: systolic and diastolic pre-dialysis BP of the deceased ones, respectively.

**Figure 2:**
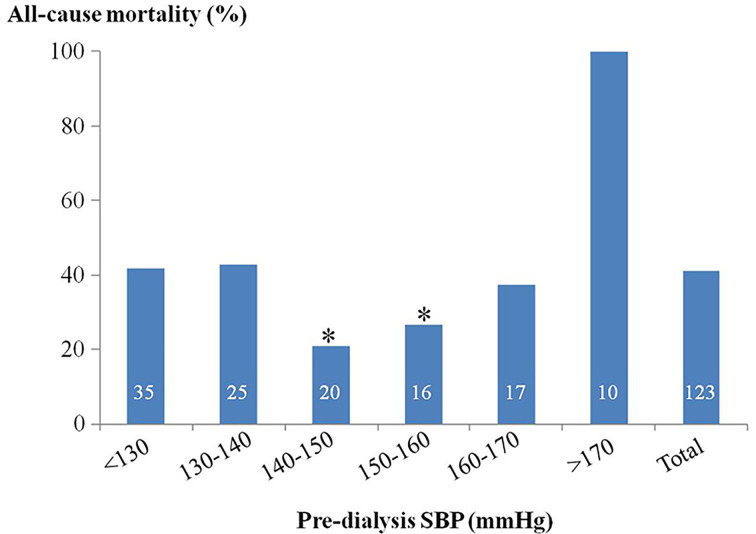
Percentage of all-cause mortality in MHD patients of 60–80 years as stratified by the pre-dialysis SBP The number on individual column indicates the patients in each group. *P<0.05 compared with other groups by ANOVA.

**Figure 3:**
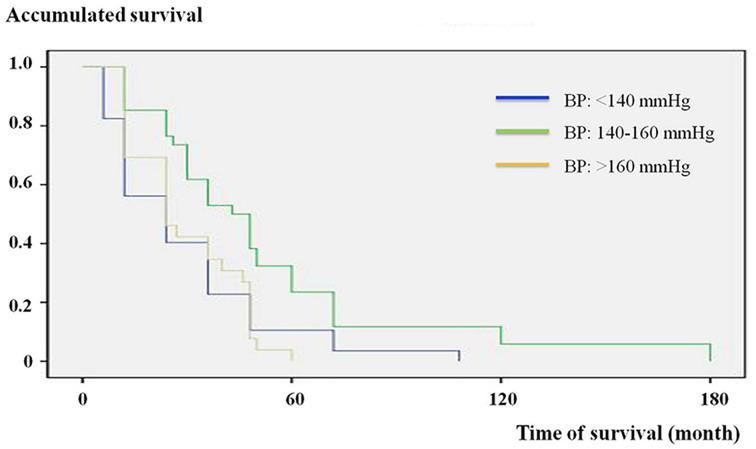
Kaplan–Meier analysis of survival based on pre-dialysis SBP in MHD patients of 60-80 years All-cause mortality was lower in the subgroup with the said BP between 140 and 160 mmHg. *P*<0.05 by log rank test.

The pre-dialysis BP of each individual was then marked on the scatterplot on which the x-axis was age (Figure 1). The distribution of pre-dialysis SBP showed clear intergroup deviation in patients of 60–80 years. Within this said age group, other significantly different variables were Scr, hs-CRP, ferritin content with the addition of FBS and iPTH, whereas the diabetic status turned out to be the only one showing a tendency (Table 2). Subsequently, multiple logistic regression successfully confirmed the above variables except the ferritin content and iPTH as independent determinants of mortality. Furthermore, a pre-dialysis SBP of 140–160 mmHg in these patients had significant minimum all-cause mortality (23.5%) against that conferred by either a lower (42.1%) or higher SBP value (61.5%) (Figure 2). These findings were then cross-validated by the Kaplan–Meier survival analysis (Figure 3). Finally, patients lost to CVD deaths in this age group (*n*=35, 70.0%) was significantly higher than that in the whole study population (49.4%). With similar process but different results, the significant determinants for CVD deaths were pre-dialysis SBP, Scr, hs-CRP, Hb and iPTH. Due to the relatively small number, we did not attempt to further pursue the optimal BP and associated survival curve for CVD deaths.

**Table 2:** Demographic and clinical profile of the subjects between 60 and 80 years

	Deceased	Surviving
*n*	50	73
Gender (male/female)	28/22	35/38
Age (y)	71.7 ± 6.4	70.2 ± 6.5
Time on dialysis (m)^‡^	46.8 ± 17.8	43.5 ± 17.3
Body mass index (kg/m^2^)	22.3 ± 3.7	22.8 ± 3.2
Pre-dialysis SBP (mmHg)	147.7 ± 30.4	136.8 ± 19.9*
Pre-dialysis DBP (mmHg)	75.9 ± 11.5	70.2 ± 13.8
Hb concentration (g/l)	90.2 ± 11.7	99.1 ± 11.1
Pre-dialysis Scr (μmol/l)‡	568.7 ± 192.5	707.5 ± 191.3*
Fasting blood sugar (mmol/l)^‡^	6.6 ± 3.9	5.9 ± 1.8*
hs-CRP (mg/l)^‡^	21.1 ± 7.5	5.9 ± 6.6*
Total cholesterol (mmol/l)	3.68 ± 1.13	4.04 ± 1.26
High-density lipoprotein (mmol/l)	1.00 ± 0.39	1.06 ± 0.41
Low-density lipoprotein (mmol/l)	2.36 ± 0.95	2.75 ± 1.00
Triglycerides (mmol/l)^‡^	1.29 ± 0.75	1.40 ± 1.06
Albumin (g/l)	30.7 ± 6.2	35.2 ± 6.1
Kt/V	1.26 ± 0.26	1.27 ± 0.26
Potassium (mmol/l)	4.7 ± 0.9	4.6 ± 0.7
Calcium (mmol/l)	2.1 ± 0.2	2.1 ± 0.2
Phosphate (mmol/l)	1.7 ± 0.7	1.8 ± 0.6
Parathyroid hormone (pg/ml)^‡^	46.2 ± 27.2	40.4 ± 20.4*
Ferritin (ng/ml)^‡^	197.7 ± 110.1	301.3 ± 134.7*
TSAT (%)	27.0 ± 19.5	26.2 ± 11.0
Diabetes mellitus (%)	58.0	41.1†

Results are given as mean ± SD. Differences among the cohorts were examined by the *t* test or χ^2^ test when deemed appropriate.

* *P*<0.05.

† 0.05<*P*<0.10.

‡ Log-transformed values used in the analysis.

## Discussion

Survival on hemodialysis is dismal that the 5-year survival was only 40% in a large prospective study from America, with 40% of the deaths being cardiac [17]. These gloomy facts were consistently found in our study: the average time on MHD was 40.3 ± 13.5 months for the deceased patients before 49.4% of them succumbed to CVD deaths. Our ultimate answer to these challenges was the discovery of pre-dialysis SBP in the range of 140–160 mmHg with the minimum all-cause mortality in the form of a U-shaped association among a Chinese MHD cohort. *A priori*, finding of this optimal BP may greatly contribute to the clinical practice in numerous hemodialysis centers in our region and enormously benefit a large number of cognate MHD patients. Upon and beyond these findings, other risk factors of mortality were further unraveled in an integrated approach.

The concept of reverse epidemiology could not be circumvented when dealing with the survival of MHD patients [18]. It refers to the paradoxic observations that the risk factors associated with increased mortality, including a higher risk of CVD events and deaths in MHD patients are different from those in the general population. In terms of the BP, hypertension is prevalent in 75–80% of the MHD patients and it remains the most controversial prognostic marker for these patients [19]. In contrast with the guidelines for the general population, in which SBP ≤ 120 mmHg is considered normal, the ideal target BP in MHD patients still lacks a consensus [20]. Neither is the type of BP measurement with respect to mortality conclusive. These disparities may indicate the involvement of ethnical, geographical and socioeconomic variations. Conceivably, a specific MHD cohort may need a distinctive optimal BP for their very survival. Similar to our findings, a U-shaped association between the optimal SBP and all-cause mortality was reported previously [21]. In this report of French MHD patients, however, a pre-dialysis SBP with nadir of 165 mmHg conferred the lowest hazard ratio of all-cause mortality, against the corresponding value of 160–189 mm Hg in American [22] and 150 mmHg in our study. By essence, this difference may be partly due to the aforementioned ethnic heterogeneity *per se*. Further in the realm of the reverse epidemiology, the role of the malnutrition-inflammation-atherosclerosis (MIA) syndrome is equally important to the MHD patients’ outcomes [23].

The namesake components of the MIA syndrome have been known for decades as predictors of a short life expectancy for the MHD patients [24]. Indeed, the mortality rate within 2 years in an Indian MHD cohort was 0, 8.7, 13.0 and 45.0% when none, one, two and three of these components were present, respectively [25]. In general agreement, we found that the pre-dialysis Scr, hs-CRP and pre-dialysis SBP were significant determinants of all-cause mortality as each may serve as a surrogate marker for the individual component of the MIA syndrome [26]. The list of determinants was further expanded to comprise the iPTH and Hb in case of the CVD deaths. Undoubtfully, the iPTH is a proven CVD risk factor [27] and the deleterious effect of anemia was widely known. In a sense of trade-off disequilibrium, however, we found that aggressive pursuit of anemia control in the MHD patients may somehow aggravate the erythropoietin resistance [15] and the accumulation of ferritin secondary to anemia was cautioned by Kalantar-Zadeh et al. [28] who had also raised the crucial issue of ‘time discrepancies’ among competitive risk factors [18].

**Figure 4:**
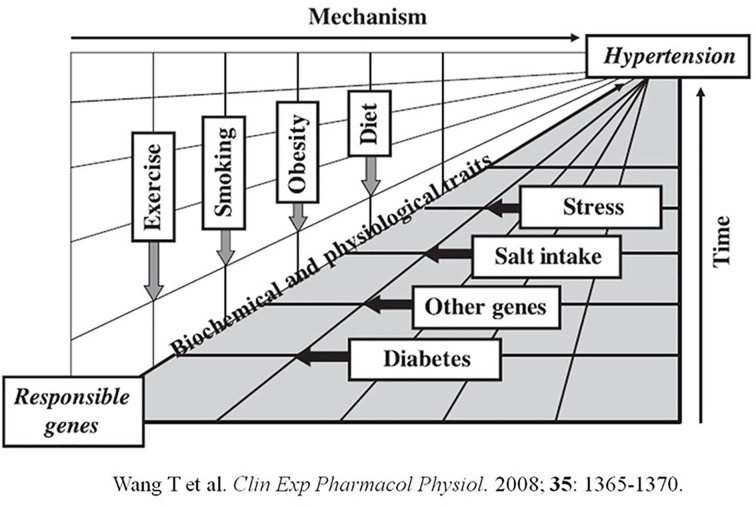
Gene–environment interactions in the development of hypertension Environmental stimuli may affect the expression of genetic information and genetic factors may, in turn, affect responses to environmental stimuli. Also presented is the ‘intermediate phenotype’ concept that certain biochemical and/or physiological traits may be ‘intermediate’ pertinent to the causality and time in the development of hypertension. This figure was used to schematically elucidate that the effect of pre-dialysis BP on all-cause mortality was also a ‘function of time’.

As for the evolution of chronic diseases, risk factors primarily exert their detrimental effects as a function of time. This notion was described schematically in our early work on the genetic variation and development of hypertension (Figure 4) [29]. A detriment may gradually progress via certain mechanism over time and, upon reaching the critical flash point, render discernible influence on the disease. Convergence of multiple detriments and/or possible compromise of the protective function may greatly exacerbate the disease progression. This may explain that our optimal pre-dialysis SBP for all-cause mortality was detected exclusively in patients of 60–80 years, who also suffered from higher CVD deaths than the whole study population. In a concordant way, BP is believed to be relevant for survival only in those MHD patients who survived long enough on dialysis to develop secondary target-organ damage, especially cardiac hypertrophy. Such damage caused by non-malignant hypertension usually requires years to become established and senior patients are more susceptible to the resultant systolic dysfunction [30]. Nevertheless, the MHD patients are considered exceptional people from the general population that they have a greater mortality risk and shorter life expectancy [31]. Not unexpected, the long-term effects of traditional risk factors on future mortality may be outweighed or even reversed by their short-term effects on dialysis mortality. Thus, we believe that rigorous BP control in MHD patients may be less relevant or perhaps harmful. Needless to say, both function of time and time discrepancy should be taken into account in the practice of MHD.

Following these lines of evidence, there should be less surprise in the disassociation of pre-dialysis DBP with all-cause mortality. In fact, the pre-dialysis DBP had been found to manifest either no independent prognostic value in many studies [6,9] or an L-shaped curve for this said mortality [21]. Under the circumstance of MHD, the pre-dialysis SBP may exhibit the typical direct association between BP and mortality as a proxy for the enhanced sympathetic activation or accelerated arterial aging thus stiffness [32], whereas the pre-dialysis DBP does not seem to suffice especially when the volume overload is handled properly. Nonetheless, it should be recognized that for any given SBP in a patient on MHD a lower DBP magnifies the risk for future mortality. Frankly, strength of our study was derived from the long observation time and main limitation was the relatively small patient number. Hence, prospective study enrolling large number of MHD patients is warranted, to further pursue the role of DBP in all-cause mortality and CVD deaths.

## Conclusion

Our report provides evidence for a U-shaped association between the pre-dialysis SBP and all-cause mortality in a Chinese MHD cohort of 60–80 years, in which the effect of BP on mortality appeared to be a function of time. Further, concerted action of mechanisms represented by the pre-dialysis SBP, hs-CRP and Scr may mimic that of MIA syndrome. Our findings may comprehensively help to focus on but not limit to the optimal BP to achieve better survival in these patients.

## Abbreviations

BP: blood pressure
CVD: cardio-cerebrovascular disease
DBP: diastolic BP
ESRD: end stage renal disease
FBS: fasting blood sugar
Hb: hemoglobin
hs-CRP: high sensitivity C-reaction protein
iPTH: intact parathyroid hormone
KDIGO: Kidney Diseases Improving Global Outcome
MHD: maintenance hemodialysis
MIA: malnutrition-inflammation-atherosclerosis
SBP: systolic BP
Scr: serum creatinine
TSAT: transferrin saturation
